# Autoimmune Thyroiditis in Patients With Diabetes Mellitus: Retrospective Study of 91 Cases

**DOI:** 10.7759/cureus.63805

**Published:** 2024-07-04

**Authors:** Amal Hanafi, Wiame Lakhlili, Mounia Bouabdellah, Laïla Benchekroun

**Affiliations:** 1 Central Laboratory of Biochemistry, Faculty of Medicine and Pharmacy, Ibn Sina University Hospital, Mohammed V University, Rabat, MAR; 2 Pharmacy Inspection and Sector Monitoring Department, Directorate of Medicine and Pharmacy (DMP) Ministry of Health and Social Protection, Rabat, MAR

**Keywords:** anti-thyroglobine antibody, anti-thyroid peroxydase antibody, thyroid antibodies, diabetes mellitus, autoimmune thyroiditis

## Abstract

Background

Detection and quantification of anti-thyroid antibodies make it possible to confirm the diagnosis of thyroid dysfunction as well as its autoimmune origin and monitor thyroid damage in diabetic patients. The aim of this study is to determine the seroprevalence of anti-thyroid antibodies in hospitalized diabetic patients.

Materials and methods

This retrospective study focused on 91 diabetic patients hospitalized in the endocrinology department of Ibn Sina Hospital, Rabat, Morocco, between January 1 and December 31, 2022. The study population was divided into two groups: 19 patients with type 1 diabetes (13 females and six males, with an age range of 20-70 years) and 72 patients with type 2 diabetes (52 females and 20 males, with an age range of 40-71 years). Hemoglobin (HbA1c) levels were determined with high-performance liquid chromatography (Hb-HPLC) analyzer from blood samples collected in EDTA tubes, and anti-thyroid antibodies (anti-TPO and/or anti-TG) were measured by chemiluminescent microparticle immunoassays (CMIA) in human serum using the ALINITY analyzer.

Results

Among type 1 diabetic patients, 42.1% (n = 8)* *were positive for anti-TPO and anti-TG antibodies, while 31.5% (n = 6)* *were positive only for anti-TPO antibodies. Among type 2 diabetic patients, 15.2% (n = 11) were positive only for anti-TPO antibodies, while 20.8% (n = 15) were positive for anti-TPO and/or anti-TG antibodies. The prevalence of anti-thyroid antibodies was higher in females, consistent with other studies. This could be linked to the involvement of autoimmune processes in the development of thyroid dysfunction in type 2 diabetics.

Conclusions

Testing for anti-thyroid antibodies in diabetic patients and their relatives helps detect subclinical conditions, which could later manifest as biological and clinical deficiencies, guiding monitoring parameters.

## Introduction

Autoimmune diseases (AIDs) encompass a broad range of conditions that target different parts of the body. A Moroccan study of 3,182 cases aimed to explore the prevalence and characterize the autoantibody profiles of different AIDs; it reported that 30% of cases (n = 955) suffered from AIDs, including autoimmune thyroiditis and type 1 diabetes [1]. These two AIDs, characterized by the infiltration of T and B lymphocytes into the gland, seem to share a common genetic origin, and their relationship is well-known and documented [2,3]. However, the relationship between thyroid dysfunction and type 2 diabetes mellitus (DM) is not yet sufficiently clarified, although studies increasingly demonstrate a connection between them [3].

It is sometimes difficult to diagnose thyroid abnormalities in diabetics based on the patient’s clinical picture because symptoms of hyperthyroidism can mimic symptoms of hyperglycemia, such as weight loss despite increased appetite and tiredness. The same hypothyroidism could be confused with the development of diabetic nephropathy since the patient may present with weight gain, edema, pallor, and easy fatigability [3]. The detection and quantification of anti-thyroid antibodies have shown that they are useful in thyroid dysfunction to confirm the autoimmune origin or to monitor thyroid damage in diabetic patients. The aim of this study is to determine the seroprevalence of anti-thyroid antibodies among hospitalized patients with diabetes.

## Materials and methods

Study design and population

This retrospective study spans one year between January 1 and December 31, 2022, involving 91 diabetic patients hospitalized in the endocrinology department of Ibn Sina Hospital, Rabat, Morocco. Their glycated hemoglobin A1C (HbA1c) levels and antibodies anti-thyroid peroxidase (anti-TPO) and/or anti-thyroglobulin (anti-TG) were measured at the central biochemistry laboratory of Rabat University Hospital.

The study population was divided into two groups. The first group comprised 19 type 1 diabetic patients, including 13 females and six males, aged between 20 and 70 years. The second group consisted of 72 type 2 diabetic patients, with 52 females and 20 males, aged between 40 and 71 years.

Laboratory measurements

HbA1c was determined with high-performance liquid chromatography (Hb-HPLC) analyzer based on reverse-phase cation-exchange chromatography (ARKRAY ADAMS A1c HA-8180T analyzer) from blood samples collected in EDTA-potassium tubes.

Anti-TPO and anti-TG antibodies were measured by chemiluminescent microparticle immunoassays (CMIA), which allows the quantitative measurement of Ig class antibodies against thyroperoxidase (anti-TPO) and thyroglobulin (anti-TG). These tests can be performed on serum collected in dry tubes or plasma collected in EDTA, lithium heparin, or sodium heparin tubes using the Alinity analyzer.

Diagnostic criteria

The study involved adult patients hospitalized in the endocrinology department of Ibn Sina Hospital with type 1 or type 2 DM. Glycated HbA1c levels were measured concurrently with anti-thyroid antibody assays in the central biochemistry laboratory of the same hospital. Exclusion criteria included individuals who did not have diabetes and those who had not received glycated HbA1c or anti-thyroid antibody testing.

This approach aligns with literature indicating that HbA1c is a critical marker for long-term glycemic control, with major clinical trials showing a strong correlation between HbA1c levels and clinical outcomes in both types of diabetes [4]. The International Expert Committee has established that an HbA1c level of 6.5% (48 mmol/mol) is diagnostic of type 2 diabetes [5].

Higher levels than 4.11 IU/mL (with a confidence interval of 97.8%) for anti-TG antibodies and 5.61 IU/mL for anti-TPO antibodies (with a confidence interval of 97.8%) were considered as criteria for inclusion.

The methods used provide us with positivity thresholds as follows (Table 1):

**Table 1:** Interpretation of thyroid and hemoglobin A1C test results

Positive results	Negative results
Anti-thyroid peroxidase antibody (≥5.61 IU/mL)	Anti-thyroid peroxidase antibody (<5.61 IU/mL)
Anti-thyroglobulin antibody (≥4.11 IU/mL)	Anti-thyroglobulin antibody (<4.11 IU/mL)
Hemoglobin A1C (HbA1c) (≥6.5%) (≥48 mmol/mol)	Hemoglobin A1C (HbA1c) (<6.5%) (<48 mmol/mol)

Data collection and analysis

Patient data included variables such as a permanent patient identifier (PPI), gender, age, and clinical information, including type of diabetes (type 1 or type 2 DM), glycated HbA1C level, anti-thyroglobulin antibodies (ATG), and anti-thyroid peroxidase antibodies (ATPO). These variables were collected using structured data collection forms and entered into Microsoft Excel® version 2013. Descriptive and statistical analysis was performed using Jamovi®.

## Results

In the first analysis, which examined the prevalence of anti-thyroid antibodies (AAT) among 19 patients with type 1 diabetes, it was found that 16 of 19 patients tested positive for anti-thyroid antibodies. Specifically, it was found that 42.1% (n = 8) of the participants tested positive for both anti-ATPO and anti-ATG, and anti-ATPO alone was detected in 31.5% (n = 6) of the patients with type 1 diabetes (Table 2).

**Table 2:** Seroprevalence of anti-thyroid antibodies in type 1 and type 2 diabetics

	«Anti-thyroid peroxidase antibody» only	«Anti-thyroglobulin antibody» only	«Anti-thyroid peroxidase antibody» and/or «anti-thyroglobulin antibody»
Type 1 diabetes (n = 19)	6 (31.5%)	2 (10.5%)	8 (42.1%)
Type 2 diabetes (n = 72)	11 (15.2 %)	6 (8.3%)	15 (20.8%)

In the second analysis, we investigated the prevalence of AAT in 72 patients with type 2 diabetes, and it was found that 32 of 72 patients tested positive for anti-thyroid antibodies. Specifically, it was revealed that 15.2% (n = 11) of the patients identified positive for anti-ATPO. Additionally, anti-ATG and/or anti-ATPO were in 20.8% (n = 15) of the patients with type 2 diabetes (Table 2).

## Discussion

DM and thyroid dysfunction are frequently observed as endocrine diseases in adults. Insulin and thyroid gland hormones work together synergistically to regulate cellular metabolism. Disruptions in the levels of either hormone can lead to dysregulation of the other group of hormones [6]. This interaction made us wonder about the association between DM and AIT. Although our study was limited by the small size of our study population, which represented all patients hospitalized with AIT between January 1 and December 31, 2022, the statistically significant observations demonstrate the importance of continuing this study over a longer period and with a larger number of patients by introducing, for example, other hospital centers in the kingdom of Morocco.

Type 1 diabetes and AAT

Compared to subsequent studies on AAT seroprevalence in type 1 diabetics, the percentage of AATPO positivity alone (31.5%, n = 6) was similar to that reported in the Tunisian study (37%, n = 13) (Table 3) and the American study (26%, n = 211), but it is higher than that (7%, n = 7) reported in the Greek study [7-9].

**Table 3:** Frequency of anti-thyroid antibodies in type 1 diabetic patients compared to literature data [7-9]

	Étude américaine (n = 814)	Étude du grec (n = 97)	Etude tunisienne (n = 35)	Notre étude (n = 19)
«Anticorps anti-thyroïde peroxydase» positif seulement (%)	26	7	37.14	31.5
«Anticorps anti-thyroglobuline» positif uniquement (%)	17	3	11.42	10.5
«Anticorps anti-thyroïde peroxydase» et/ou «anticorps anti-thyroglobuline» (%)	29	89	39	42.1

Type 1 DM is an AID characterized by the destruction of insulin-producing β-cells (insulocytes) in the pancreas by autoreactive T cells and autoantibodies. This leads to insulin deficiency, resulting in insulin resistance and hyperglycemia [10]. Type 1 DM shares a potential genetic basis with other autoimmune endocrine disorders, such as autoimmune thyroid disease (AIT), due to their frequent co-occurrence within families and individuals. Genetic susceptibility loci associated with these conditions reported in the literature include the cytotoxic T-lymphocyte associated protein 4 (CTLA4), protein tyrosine phosphatase non-receptor type 22 (PTPN22), and human leukocyte antigen class II (HLA-II) [9-12].

It appears that age and female sex may significantly influence the co-occurrence of autoimmune thyroiditis (AIT) and type 1 DM. An Australian study reports a low seroprevalence of AATPO positivity, not exceeding 8%. However, it highlights an increase in this percentage with the duration of diabetes [13]. Specifically, the 8% positivity was observed at the time of autoimmune type 1 diabetes diagnosis (in patients aged 0-15 years), and a follow-up after seven years showed an increase to 11.6%. Thus, the age of onset of the autoimmune condition could explain the high prevalence of AATPO in our population of type 1 diabetics, which is predominantly composed of adults (aged over 14 years). The duration since the onset of the disease was not considered in our study [13].

Research from Germany and Austria involving children with type 1 diabetes revealed that 22% (n = 1530 of 7097) displayed elevated thyroid antibodies, with females constituting 63% of those affected [12]. Moreover, a recent review has proposed that estradiol can expedite AID progression via the T-lymphocyte pathway [14]. In our own study focusing on adults aged 20-71 with type 1 diabetes, excluding pediatric cases, we found that 68% of female patients (n = 16) developed AIT, in contrast to 32% (n = 6) of male participants.

Other studies indicate that autoimmune thyroiditis can be triggered by subcutaneous administration of insulin-containing genapol© (polyethylene-polypropylene glycol) [15].

Type 2 diabetes and AAT

Our study, which included 72 type 2 diabetic participants, showed that 15.2% (n = 11) had only anti-thyroid peroxidase antibody (anti-TPO), a lower percentage compared to the Omanian study (20%, n = 20) and Tunisian study (22.5%, n = 13). The presence of only anti-thyroglobulin antibody in our study was 8.3% (n = 6), closely matching the Omanian study (9%, n = 9) and significantly higher than the Tunisian study (3.5%, n = 2). When considering the presence of either “anti-TPO” or “anti-TG” antibodies, our study found a prevalence of 20.8% (n = 15), which was similar to the Tunisian study (20%, n = 11) and lower than the Omanian study (29%, n = 29). These results indicate that the prevalence of thyroid antibodies in our population is largely consistent with the other studies (Table 4) [8,16].

**Table 4:** Comparative frequencies of anti-thyroid antibodies in type 2 diabetic patients [8,16]

	Omanian study (n = 100)	Tunisian study (n = 57)	Our study (n = 72)
«Anti-thyroid peroxidase antibody» positive only (%)	20	22.5	15.2
«Anti-thyroglobulin antibody» positive only (%)	9	3.5	8.3
«Anti-thyroid peroxidase antibody» and/or «anti-thyroglobulin antibody» (%)	29	20	20.8

We observed a higher prevalence of AATs in female type 2 diabetics, consistent with findings from similar investigations [17,18]. Studies highlight an autoimmune origin in type 2 diabetes, as demonstrated by the detection of anti-glutamic acid decarboxylase (GAD 65) antibodies. GAD 65 serves as the most significant marker of the autoimmune origin of Langerhans β-cell destruction in type 1 diabetes, being detected early in 70-90% of cases. This antibody is also found in 10-20% of type 2 diabetics [8].

Anti-thyroid antibodies (AAT): type 1 versus type 2 diabetic patients

The thyroid gland is part of the endocrine system and can be impacted by prolonged hyperglycemia and the body’s ongoing efforts to correct carbohydrate imbalances. The literature indicates a coexistence of diabetes and thyroid dysfunction, with thyroid disorders influencing glucose metabolism and untreated thyroid issues complicating diabetes management [19].

Comparatively, type 1 diabetic patients show higher percentages of anti-TPO than type 2 diabetic patients (31.5% vs. 15.2%). Similarly, type 1 diabetic patients showed higher percentages of ATG than type 2 diabetic patients (10.5% vs. 8.3%). With regard to combined ATPO and/or ATG antibodies, type 1 diabetic patients also showed higher percentages than type 2 (42.1% vs. 20.8%) (Table 2). The Tunisian and Omanian studies confirm these results, showing that type 1 diabetic patients have higher percentages of anti-thyroid antibodies than type 2 [5].

The close link between diabetes and thyroid disease has encouraged the American Diabetes Association (ADA) to recommend that people with diabetes should be checked periodically for thyroid dysfunction [20]. Thyroid disease should be screened annually in diabetic patients to detect asymptomatic thyroid dysfunction [21].

## Conclusions

At Ibn Sina University Hospital, a study spanning one year documented 48 cases of autoimmune thyroiditis among diabetic patients (n = 91). These individuals, predominantly elderly women, tested positive for anti-TG or anti-TPO antibodies, or both. This finding highlights the prevalence of thyroid autoimmunity in this demographic, highlighting the clinical importance of diabetes management and thyroid health.

Detection of anti-thyroid antibodies in diabetics, particularly type 1 diabetics and their relatives, has crucial implications for early intervention. By identifying these antibodies, healthcare providers can proactively monitor for asymptomatic hypothyroidism and other thyroid dysfunctions before they manifest clinically. This proactive approach not only helps preventatively address potential health complications but also serves as a predictive tool in scenarios such as pregnancy or when patients are undergoing specific medication treatments. Thus, integrating thyroid antibody screening into diabetic care protocols can significantly enhance patient outcomes by guiding timely interventions and tailored monitoring strategies.
